# Warner’s Sugar-Coated Pills

**Published:** 1881-07

**Authors:** 


Warner's Sugar-coated Pills.—Those of our physicians who visited the excellent exhibit of drugs and pharmaceutical preparations at the Druggists' convention last month, cannot have failed to notice the elegant display of sugar-coated pills and granules, made by Warner & Co. We have of late usually prescribed by preference gelatin sugar-coated pills, and were not disposed to give the old-fashioned sugar-coated pills much attention. The statement of a pharmacist present that a sugar-coating, properly applied. was more soluble than gelatin, prompted an investigation. That the coating was very quickly dissolved in the mouth, was promptly demonstrated, and upon cutting open several pills of different composition, the interior was found to be soft or in an easily soluble condition. We have since frequently prescribed them with very satisfactory results in every case. The appearance of sugar-coated pills is to many patients much more agreeable than the gelatin, and other things being equal, we ourselves prefer them.

